# Phylogenetic and Ontogenetic View of Erythroblastic Islands

**DOI:** 10.1155/2015/873628

**Published:** 2015-10-18

**Authors:** Katie M. Giger, Theodosia A. Kalfa

**Affiliations:** Cancer and Blood Diseases Institute, Cincinnati Children's Hospital Medical Center, University of Cincinnati College of Medicine, Cincinnati, OH 45229, USA

## Abstract

Erythroblastic islands are a hallmark of mammalian erythropoiesis consisting of a central macrophage surrounded by and interacting closely with the maturing erythroblasts. The macrophages are thought to serve many functions such as supporting erythroblast proliferation, supplying iron for hemoglobin, promoting enucleation, and clearing the nuclear debris; moreover, inhibition of erythroblastic island formation is often detrimental to erythropoiesis. There is still much not understood about the role that macrophages and microenvironment play in erythropoiesis and insights may be gleaned from a comparative analysis with erythropoietic niches in nonmammalian vertebrates which, unlike mammals, have erythrocytes that retain their nucleus. The phylogenetic development of erythroblastic islands in mammals in which the erythrocytes are anucleate underlines the importance of the macrophage in erythroblast enucleation.

## 1. Introduction

**Figure 1:**
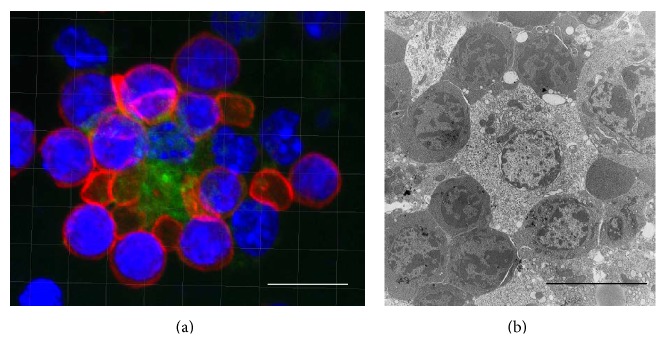
Imaging of erythroblastic islands. (a) Confocal immunofluorescence image of adult mouse bone marrow. The long bones were flushed and the marrow gently dispersed and fixed before staining with AF488-conjugated F4/80 (green) and AF647-conjugated Ter119 (red) antibodies and DAPI for nuclear stain (blue). Scale bar 10 *μ*m. (b) Transmission electron microscopy (TEM) image of an erythroblastic island in an E14 mouse fetal liver. Scale bar 10 *μ*m.

The terminal differentiation of mammalian erythroblasts to produce red blood cells (RBCs) occurs in specialized niches of the fetal liver, bone marrow, and spleen called erythroblastic islands (Figure 1). Florence Rena Sabin, Professor of Histology in the Johns Hopkins Medical School and the first woman to be elected in the United States National Academy of Sciences, described almost a century ago the origin of the red blood cells from erythroblasts in the chicken yolk sac and rabbit bone marrow [1]. In her microscopy images of bone marrow histological sections, she notes that the erythroblasts are arranged “in groups” [2]. Bessis, French hematologist and researcher, demonstrated first in 1958 using electron microscopy that the groups of erythroblasts surround a central macrophage and portrayed the erythroblastic island (EBI) as the erythropoietic niche where erythroblasts mature and eventually are enucleated to produce reticulocytes [3].

These islands in the hematopoietic tissues of rodents and humans have since been the subject of several studies and of excellent reviews [4–6] but their precise role in erythropoiesis is still under debate. The macrophages may provide iron for hemoglobin synthesis and growth signals to regulate erythroblast proliferation and survival and likely play a role in enucleation of both primitive and definitive erythrocytes. The erythroblast-macrophage interaction has been demonstrated to be an important factor for successful erythropoiesis both* in vivo* and* in vitro* and understanding the role of the macrophage in this process is important for improving* in vitro* culture systems for mass production of RBCs to be utilized as transfusion resources. Insights may be found by looking at requirements for erythropoiesis in other species, especially those in which mature erythrocytes are not enucleated. In this review, we will discuss the role that macrophages and erythroblastic islands may play in erythropoiesis along mammalian development and across the animal kingdom.

## 2. Ontogeny of Erythroblastic Islands in Mammals

The initial wave of “primitive” RBC production originates in the embryonic yolk sac. In mouse embryos, erythroid precursors still immature enter the bloodstream as vessels are created in embryonic day 8.25 (E8.25) soon after the onset of cardiac contractions and differentiate as a semisynchronous cohort while in circulation [7, 8]. A second transient wave of “definitive” erythroid progenitors from the yolk sac also enters the bloodstream and seeds the liver of the fetus. In parallel, at ~E10.5, hematopoietic stem cells from multiple sites within the embryo, including the para-aortic splanchnopleura, the aorta-gonad-mesonephros (AGM) region, other large arteries (vitelline and umbilical), and the placenta, also seed the liver [7, 9–12]. The fetal liver is recognized as the first site of adult-type “definitive” erythropoiesis, and it is the first site where erythroblastic islands with a central macrophage are identified. Sequentially, postnatally in mice or during the second trimester of intrauterine life in humans, EBIs are found throughout the bone marrow in mammals, the hematopoietic tissue where homeostatic adult erythropoiesis takes place. They also appear within the red pulp of the spleen and other sites of extramedullary hematopoiesis [13, 14] indicating that the microenvironment they comprise is important not only for steady state, but also for stress erythropoiesis.

Although it was once thought that EBIs were located near the sinusoids for convenient egress of reticulocytes into the circulation analogous to the positioning of megakaryocytes to facilitate platelets entrance [15, 16], detailed ultrastructural studies have shown that EBIs are actually distributed throughout the marrow as well as the fetal liver [17–19]. In normal homeostatic bone marrow, erythroblasts in a given island are typically of various differentiation stages. However, with a brilliant experimental design of suppression of erythropoiesis in rats with hypertransfusion and subsequent stimulation with exogenous erythropoietin (EPO), Mohandas and Prenant showed by EM studies in serial sections that the erythroblasts within an island may arise from a common precursor and mature as discrete synchronized clusters which cannot be observed in the more densely packed steady-state bone marrow [17]. Yokoyama et al. observed in EM studies of rat marrow that orthochromatic erythroblasts were found more frequently at EBIs near the sinusoids and that proerythroblasts were often found further away [18]. Based on their observations, they propose that erythroblastic islands form away from the sinusoid and either the whole island or only the erythroblasts migrate towards the sinusoid as erythroid maturation proceeds [18] though this hypothesis has not yet been confirmed experimentally.

The role that erythroblastic islands and the central macrophages play in erythropoiesis is not completely understood and yet it is clear from decades of studies that functional interaction between erythroblasts and macrophages is indispensable for optimal erythroid maturation and enucleation. One long-suspected role of the central macrophages is that they export ferritin which is taken up by the erythroblasts and used for hemoglobin synthesis [20]; this was recently demonstrated in a transferrin-free human coculture system [20, 21]. Although it is still unclear how much this function contributes to erythropoiesis* in vivo*, increasing evidence is accumulating on the role of “nurse” macrophage in iron trafficking towards the maturing erythroblasts [22].

Erythroid differentiation at islands is regulated via a number of mechanisms including the release of soluble factors which mostly exert paracrine effects and through direct cell-cell interactions. Macrophages secrete a number of factors which negatively regulate erythropoiesis including IL-6, TGF-*β*, TNF-*α*, and INF-*γ* [23–25]; alternatively, they can promote BFU-E and CFU-E growth through secretion of insulin-like growth factor and erythroid burst-promoting activity (BPA) [4, 26]. In response to EPO, erythroblasts secrete Gas6 which enhances survival response to EPO receptor signaling and decreases inhibitory signaling from the macrophages [27]. Erythroblasts have also been shown to secrete angiogenic factors VEGF-A and PDGF which may modulate erythroblast interactions with the endothelium to facilitate their egress from the niche [28]. Direct cell-cell interactions also regulate erythropoiesis within the islands. The expression of death receptor ligands on more mature erythroblasts leads to caspase-mediated degradation of GATA-1 which inhibits the expansion and differentiation of immature erythroblasts [29, 30]. Alternatively, adherence of erythroblasts to macrophages has been shown to increase erythroblast proliferation and decrease apoptosis [31], and to this end, the central macrophages are always observed to be in close contact with the erythroblasts often cupping them with thin cytoplasmic extensions [17, 18]. Several surface receptors are known to mediate these interactions. Integrin *α*_4_ *β*_1_ expressed on erythroblasts mediates multiple interactions via binding with VCAM-1 on the central macrophage and ICAM4 on neighboring erythroblasts [32, 33]. Deletion of ICAM4, which is also shown to interact with macrophage integrin *a*_*V*_, resulted in decreased island formation but had little effect on steady-state erythropoiesis [33]. However, blocking *α*_4_ *β*_1_ with antibodies decreased cell proliferation and increased apoptosis* in vitro* [34] and embryos null for integrin *α*_4_ die in utero after the 12th embryonic day (E12) due in part to inefficient erythropoiesis [35]. Erythroblast Macrophage Protein (EMP or MEAE) is expressed on both the central macrophage and the maturing erythroblasts and mediates an interaction between them [36]. Blocking this interaction* in vitro* led to increased apoptosis and failed erythropoiesis [36]. It was later observed that EMP expression on the central macrophage was required for its interaction with erythroblasts while EMP on the erythroblasts appeared to be required for their enucleation [37]. The heme scavenger CD163 was shown to form a direct interaction with erythroid cells and promote their proliferation in rat and human erythroid cultures [38], but the complementary receptor on the erythroblast is not yet known.

Lastly, the central macrophages promote erythroblast enucleation and phagocytose and digest the extruded nuclei. The process of enucleation is not completely understood yet but it is proposed to be a form of asymmetric cytokinesis [39–41]. Macrophages are believed to promote the process and they significantly increase the efficiency of enucleation in murine and human erythropoiesis cultures [42, 43]. Human CD34^+^ cultures in the absence of microenvironment (i.e., stromal cells or macrophages) are generally enucleated with suboptimal efficiency, ranging from <10% to as much as 40–50% [43]. On the other hand, when cocultured with stromal cells or macrophages, nearly complete enucleation is achieved [44, 45]. Macrophages are also required to phagocytose and digest the extruded nucleus [46]. Polarization of the cell precedes enucleation and studies have confirmed sorting of red cell cytoskeletal and membrane proteins to one side while the nucleus is pushed to the other side and eventually is separated from the cell surrounded by membrane and a small rim of cytoplasm. The expelled nucleus, or pyrenocyte, is rapidly engulfed by the macrophage owing to its externalization of phosphatidylserine [47] and presence of adhesive receptors which are sorted with the nucleus [48]. Meanwhile, the consequent decrease of adhesive proteins on the nascent reticulocyte ensures successful detachment from the niche and entry into the circulation via the sinusoids. The central macrophages express high levels of DNase II which allows them to break down the nuclear material. Transgenic mice with DNase II deficiency develop fatal intrauterine anemia, associated with decreased erythroblastic island interactions, which suggests that the recycling of the nuclei is an important factor that regulates erythropoiesis in a non-cell-autonomous way [49].

## 3. Phylogeny of Erythroblastic Islands

The only reported role of the macrophage in primitive mammalian erythropoiesis appears to be assisting with the enucleation. Primitive RBCs are enucleated by nuclear extrusion to generate erythrocytes and pyrenocytes after association with macrophages in the erythroblastic islands of the fetal liver [51]. Thus, we could expect erythroblastic islands to play little or no role in erythropoiesis of nonmammalian vertebrates (birds, reptiles, amphibians, and fish) which have nucleated erythrocytes circulating throughout embryogenesis and adulthood. Some exceptions have been reported in this rule: anucleate erythrocytes have been found in some species of salamander (Plethodontidae) [52] and in one teleost fish species (*Maurolicus muelleri*) [53]. In these cases, however, much variability in size and shape of RBCs is observed along with free nuclei and evidence indicates that the anucleate erythrocytes are produced by mechanical rupture of the cells after twisting in circulation through capillaries too narrow for their nuclear size [53, 54].

Despite the primitive (nucleated) appearance of the circulating adult nonmammalian RBCs, there are also two waves of erythropoiesis in nonmammalian vertebrate embryos. In almost all vertebrates, embryonic hematopoiesis originates from the yolk sac; in the teleost species of the bony fishes (osteichthyes) embryonic erythroid cells form in a distinct dorsal-lateral compartment of the embryo known as the intermediate cell mass of Oellacher [55]. Primitive erythroid cells can be distinguished in nonmammalian vertebrates based on their larger size and expression of embryonic globins similarly to mammalian primitive erythroids. Very little has been described on the role, if any, of macrophages in erythropoiesis in nonmammalian vertebrates. Moreover, presence of erythroblastic islands with a central macrophage is not reported in nonmammalian embryos or adult organisms. We will account briefly here for the topography of erythropoiesis in the ontogeny of nonmammalian vertebrates in order to point to some interesting differences and similarities with mammalian erythropoiesis.

Avian erythropoiesis initiates in the yolk sac and then sequentially moves to the liver and spleen and then to the adult bone marrow. The bone marrow erythropoiesis occurs within the sinusoids, while granulopoiesis occurs within the extrasinusoidal space of the bone marrow; granulocyte precursors are never seen with the erythroblasts (also known as rubriblasts). The sinuses are lined by endothelial cells which interact with the maturing erythroblasts, presumably keeping them from entering the circulation prematurely and possibly participating in the regulation of erythroid maturation [56]. As rubriblasts mature, the nuclear size decreases, the chromatin becomes increasingly condensed, and the amount of cytoplasm increases along with the cellular hemoglobin concentration. In avian erythrocytes the cell and nuclear shape change from round to ellipsoid in the end of terminal maturation, and there is significant decrease or complete loss of the intracellular organelles like ribosomes and mitochondria [57].

**Figure 2:**
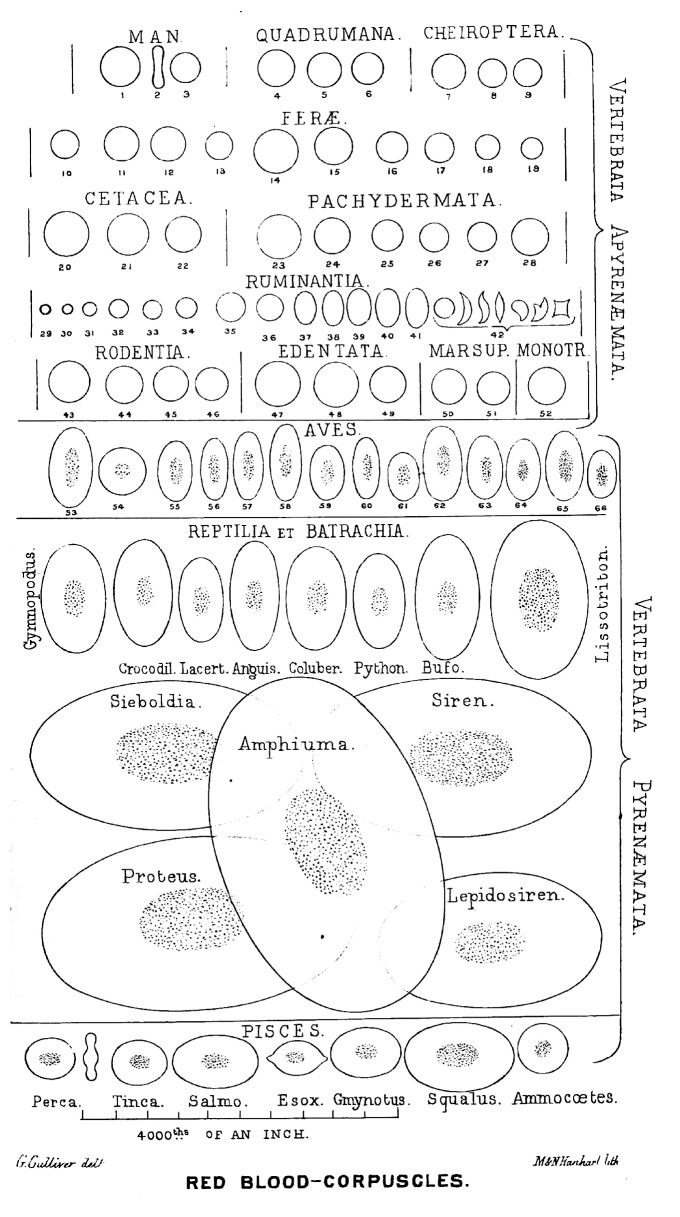
Phylogenetic view of RBC in vertebrates. This drawing is from a classic report by Gulliver (1875) [50]. (This image is in the public domain because its copyright has expired for all countries with a copyright term of life of the author plus 70 years.)

Erythropoiesis in adult reptiles also occurs within the lumen of the bone marrow sinuses, indicating that the erythroblasts mature within the marrow vascular space [58]. Of note, the mature reptilian erythrocytes are often larger than the immature erythroid precursors—a distinctive difference compared to mammals [59]. Amphibians have the largest erythrocytes in the animal kingdom (Figure 2) and, along with reptiles, have also typically longer erythrocyte lifespans than avians and mammals, which additionally increases during brumation when the metabolic activity is lower [55]. The primary site for larval erythropoiesis in most amphibians is the kidney, with the liver playing a minor role. Adult erythropoiesis mostly occurs intravascularly in the spleen, with some participation of the kidney, liver, and bone marrow [60, 61]. Bone marrow as a hematopoietic organ appears phylogenetically only in the most evolved Urodeles (amphibians with tails) and only lymphopoiesis and granulopoiesis take place there. Medullary erythropoiesis occurs in the other amphibian order, the Anurans (e.g., toad and frog), especially during heightened hematopoiesis following metamorphosis or hibernation, making this a phylogenetic turning point in the animal kingdom [60]. Maturation of the erythrocyte in the circulation is typical (especially in Urodeles) and involves a change in cell shape from round to ellipsoid which is maintained by the cytoskeleton, while proliferation of erythroid precursors in circulation can be induced by splenectomy or hemolysis [60].

About 27,000 species comprise the vertebrate group of fish. The vast majority (>26,000) of these belong to the class of bony fish (osteichthyes) and more than 600 to the cartilaginous class (chondrichthyes). The rest are 100 species of jawless fish (agnathans), the lamprey, and hagfish classes. The diversity among and even within classes limits the ability to make generalizations about this group. The best described fish species with regard to hematopoiesis is the zebrafish (*Danio rerio*), a member of the teleost group of bony fish, which has been established as a powerful animal model for the study of erythropoiesis and anemia [62]. Primitive hematopoiesis in zebrafish originates in the yolk sac and the anterior and posterior intermediate cell masses (ICMs), whereas the aorta-gonad-mesonephros- (AGM-) like region, caudal hematopoietic tissue (CHT), thymus, and pronephros (a discrete anterior “kidney” with renal interstitium entirely devoted to hematopoiesis) are the sites of definitive hematopoiesis [62]. Minor hematopoietic components are found in the liver, intestines, and thymus; there is no bone marrow or lymph nodes [55, 63]. During primitive hematopoiesis between 12 and 24 hours postfertilization, macrophages are the main cell type developing within the anterior ICM, whereas the posterior ICM develops primarily into erythroid and some myeloid cells [62]. Primitive erythroid cells are closely associated with endothelial cells [64]. Macrophage precursors from the anterior ICM migrate to the yolk sac to differentiate. Many macrophages resist the blood stream while in the yolk sac and are anchored to the underlying yolk syncytial layer or to the basal lamina of the overlying ectoderm, seemingly oriented towards the site of arrival of the proerythroblasts. Herbomel et al. showed with high-resolution time-lapse DIC video microscopy that macrophages in the yolk sac stop the proerythroblasts as they enter, touch them, and in some cases almost engulf them, apparently performing a lengthy interaction and/or inspection for approximately 1 hour, and then release them back into circulation [65]. Like the mammalian and other nonmammalian vertebrate RBCs, the fish erythrocytes as they mature accumulate newly synthesized hemoglobin which appears as amorphous, homogeneous material in the cytoplasm, and although they remain nucleated, they lose their internal organelles, including nucleoli, Golgi complex, ribosomes, mitochondria, lysosomes, and degenerated organelles [66], indicating that organelle loss is not necessarily associated with the enucleation process.

In summary, sites of hematopoiesis shift during ontogeny and phylogeny and in lower vertebrates include a wide variety of organs, from the kidney and spleen to the thymus, gonads, and the brain. In many species, blood development is often compartmentalized with erythropoiesis and thrombopoiesis occurring intravascularly and lymphopoiesis or granulopoiesis occurring extravascularly; in lower vertebrates (fish and the less evolved amphibians) this compartmentalization frequently involves different organs [55, 67]. The various hematopoietic tissues often resemble the organization and function of the mammalian bone marrow (stromal layer, reticular network, sinusoids, and even adipocytes in some cases). An impressive difference, however, is the absence of macrophages in nonmammalian erythropoiesis as a stable central component within erythroblastic islands. The single most significant difference between mammalian and nonmammalian RBCs is that mammalian RBCs have been produced after an active process of enucleation. The teleological reasons of erythroblast enucleation in mammals likely correlate with evolution demanding higher oxygen delivery due to increased aerobic metabolic demands, in organisms with relatively big genome size [54]. A strong positive relationship has been noted among vertebrates between genome size and nuclear size as well as cellular volume. This would extend to RBC volume, if the RBCs would retain their nucleus as is the case in nonmammalian vertebrates [68]. There is a reverse relationship between RBC size and metabolic rate among different groups of vertebrates: for example, amphibians display the largest genome sizes and blood cells along with a low metabolic rate while birds have relatively small genome size and RBC size with a high metabolic rate [69] (Figure 2). Enucleation allows disconnection between genome size and metabolic rate since it allows a higher surface-to-volume ratio, increased rate of gas exchange, and increased efficiency of oxygen delivery by the enucleated RBCs. The phylogenetic association of enucleation with the presence of erythroblastic islands with a central macrophage appears to support the significance of macrophage-erythroblast interaction for efficient enucleation.
